# Impact of pre-pregnancy maternal body mass index on neonatal outcomes in twin delivery: A retrospective cohort study

**DOI:** 10.12669/pjms.41.1.11135

**Published:** 2025-01

**Authors:** Lianghui Zheng, Liping Huang, Jiaoxia Liu, Ting Yao, Qiuping Liao, Lin Lin

**Affiliations:** 1 Lianghui Zheng Department of Obstetrics and Gynecology, Fujian Maternity and Child Health Hospital College of Clinical, Medicine for Obstetrics & Gynecology and Pediatrics, Fujian Medical University, Fuzhou, Fujian Province 350001, China; 2 Liping Huang Department of Obstetrics and Gynecology, Fujian Maternity and Child Health Hospital College of Clinical, Medicine for Obstetrics & Gynecology and Pediatrics, Fujian Medical University, Fuzhou, Fujian Province 350001, China; 3 Jiaoxia Liu Fujian Medical University, Fujian Province 350001, China; 4 Ting Yao Fujian Medical University, Fujian Province 350001, China; 5 Qiuping Liao Department of Obstetrics and Gynecology, Fujian Maternity and Child Health Hospital College of Clinical, Medicine for Obstetrics & Gynecology and Pediatrics, Fujian Medical University, Fuzhou, Fujian Province 350001, China; 6 Lin Lin Department of Obstetrics and Gynecology, Fujian Maternity and Child Health Hospital College of Clinical, Medicine for Obstetrics & Gynecology and Pediatrics, Fujian Medical University, Fuzhou, Fujian Province 350001, China

**Keywords:** Maternal BMI, Twin pregnancy, Neonatal outcomes, Preterm birth, Low birth weight, Retrospective cohort

## Abstract

**Objective:**

This study examined the potential link between maternal pre-pregnancy body mass index (PPBMI) with neonatal outcomes in twin pregnancies.

**Methods:**

This retrospective analysis records of 1,270 women with twin pregnancies, delivered at the Fujian Maternity and Child Health Hospital between 2019 and 2021, were retrospectively analysed. Women were diagnosed as underweight, normal BMI, and overweight/obese according to their PPBMI. Neonatal outcomes included low birth weight (LBW), intrauterine growth retardation (IUGR), prematurity, neonatal intensive care unit (NICU) admission, neonatal mortality, and low APGAR scores. Univariate and multivariable regression analyses were performed.

**Results:**

The proportions of neonates with LBW, IUGR, prematurity, and NICU admission were 34.7%, 2.5%, 27%, and 30%, respectively. Five neonatal deaths occurred, all born to mothers with normal BMI. Low APGAR scores at five minutes were observed in one neonate in the maternal overweight/obese group and one neonate in the normal maternal BMI group. Multivariate regression analysis showed that underweight women had an approximately twice higher risk of delivering LBW twins (OR 1.88, 95% CI: 1.26, 2.89) than women with normal BMI. No statistically significant associations were found between maternal BMI and other neonatal outcomes.

**Conclusion:**

Low PPBMI increases the risk of delivering LBW twins, independent of other factors. Our findings emphasize the importance of pre-pregnancy nutritional status in optimizing neonatal outcomes in twin deliveries.

## INTRODUCTION

Twin pregnancies represent nearly two to four percent of all births globally.^1^ In Southeast and Southern Asia, the prevalence of twin births is lower, with less than 10 twin pregnancies per 1,000 births,^2^,^3^ while in the United States, Latin America, and Africa, the rate is higher.^2^,^3^ The incidence of twin pregnancies has been on the rise currently, especially in high- and middle-income regions.^4^ This increase could be attributed to factors such as delayed maternal age at conception, declining fertility, and the growing use of assisted reproductive technologies.^3^,^4^

Twin pregnancies present unique challenges and risks, both for the mother and the neonates.^5^,^6^ Pre-pregnancy maternal body mass index (PPBMI) is a well-established indicator of maternal health^7^ and has been shown to affect perinatal outcomes in singleton pregnancies.^8^,^9^ However, its impact on neonatal outcomes in twin deliveries remains less clear. While the rates of obesity in developed countries are on the rise, many low to middle-income countries report a higher incidence of pregnant women who are underweight.^10^,^11^ Elevated PPBMI often leads to gestational diabetes, hypertension, and preterm birth, which may have amplified effects in the context of twin pregnancies.^12^–^14^ Conversely, babies of underweight women may face different but equally significant risks, including intrauterine growth retardation (IUGR) and preterm delivery.^15^,^16^ Therefore, understanding the association between PPBMI and neonatal outcomes in twin gestations is crucial.

This study aimed to investigate the impact of PPBMI on key neonatal outcomes in twin deliveries. Our results may improve clinical management and risk stratification in twin pregnancies, ultimately enhancing maternal and neonatal health outcomes.

## METHODS

This retrospective analysis used routinely collected hospital data of women with viable twin pregnancies at >20 weeks gestation whose infants were born between 28 weeks gestation and 40 weeks gestation, admitted to Fujian Maternity and Child Health Hospital between 2019 and 2021.

### Ethics Committee:

The Ethics Committee of Fujian Maternity and Child Health Hospital approved the study with the number 2024KY198, Date: August 14^th^ 2024. Due to the study’s retrospective nature, there was no need for informed consent.

### Inclusion criteria:

- Women with viable twin pregnancies.
- >20 weeks gestation.
- Delivered between 28 and 40 weeks gestation.
- Complete medical records.

### Exclusion criteria:

- Singleton pregnancies.
- Maternal pre-pregnancy or first trimester BMI not available.

### Outcomes:

The primary outcomes of interest included risk of low birth weight (LBW, birth weight<2500 grams), prematurity (gestational age<37 weeks), and intra-uterine growth retardation (IUGR, birth weight< 10th centile for a specific completed gestational age).^17^ The secondary outcomes were neonatal mortality, low APGAR score (i.e., <7 at five minutes), and admission to the neonatal intensive care unit (NICU). If one or both babies had the outcome of interest, it was coded as ‘Yes.’ If the outcome was absent in both neonates, it was coded as ‘No’ for the analysis. Information was collected on maternal educational status, age, history of preterm birth, chronic hypertension, and parity. Women were categorized based on the PPBMI: underweight (<18.5 kg/m^2^), normal (18.5−24.9 kg/m^2^), overweight (25.0−29.9 kg/m^2^), and obese (≥30.0 kg/m^2^).^18^ Data collection included antenatal anemia (hemoglobin <11 g/dL), pre-eclampsia, gestational diabetes, and cervical insufficiency, as well as mode of conception (assisted or natural), sex of the newborn, and gestational age (measured in weeks by ultrasound). Trained medical personnel collected this data during antenatal care visits. Hypertension in pregnancy was diagnosed if a systolic blood pressure was ≥140 mm Hg and/or diastolic blood pressure was ≥90 mm Hg.^19^ Gestational diabetes mellitus was diagnosed par the International Association of Diabetes and Pregnancy Study Groups consensus panel criteria.^20^

### Statistical analysis:

R software version 4.4.1. and R Studio was used. The median and interquartile range (IQR) were reported for continuous variables, while proportions were calculated for categorical variables. Baseline characteristics were presented for the three categories based on the maternal BMI. Comparisons of the baseline characteristics among the three groups for statistically significant differences were observed using Pearson’s Chi-squared test and the Fisher’s exact test for categorical variables, and the Kruskal-Wallis rank sum test for continuous variables.^21^,^22^ A multivariable logistic regression model was used. Initially, a univariate analysis was conducted with pre-pregnancy maternal BMI as the exposure variable and each neonatal outcome as the dependent variable, recording the resulting odds ratios (OR) with 95% confidence intervals (CI). Subsequently, each potential covariate was individually added to the model, and the corresponding change in OR was noted. Covariates that caused at least a 15% change in the OR were included in the multivariable logistic model to enhance the retention of meaningful confounders.^21^,^22^ Significance was determined when P<0.05.

## RESULTS

Records of 1270 women with twin pregnancies were analyzed. The baseline characteristics of the women, stratified by their pre-pregnancy BMI, are shown in (Table-I). There were 171 (13.5%) underweight, 138 (11%) overweight/obese, and 961 (75.5%) normal-BMI women in the cohort. As the number of obese women was small (n=13), their data were combined with the overweight group. We have detected a significant intergroup difference (p<0.05) in maternal education, antenatal anemia, advanced maternal age, chronic hypertension, gestational diabetes mellitus status, and pre-eclampsia associated with different pre-pregnancy maternal BMI (Table-I). As shown by the univariate regression analysis (Table-II), the risk of LBW neonates was around two times higher in underweight mothers (OR 1.93, 95% CI: 1.30, 2.92) than in women with a normal PPBMI. No association of PPBMI with other neonatal outcomes was detected (P>0.05). The association between underweight PPBMI status and LBW in offspring was further confirmed by the multivariable regression analysis (Table-III), adjusted for significantly different baseline characteristics of the cohort (maternal education, antenatal anemia, advanced maternal age, chronic hypertension, gestational diabetes mellitus status, and pre-eclampsia), with the OR of 1.88 (95% CI: 1.26, 2.89). No statistically significant associations were detected between PPBMI and other outcomes.

**Table-I:** Baseline characteristics of the mothers based on their pre-pregnancy BMI categories.

Characteristic	Underweight, N = 171^1^	Normal, N = 961^1^	Overweight or Obese, N = 138^1^	p-value ^2^
Educational status				<0.001
Junior high school/secondary school and below	41 (24%)	273 (28%)	58 (42%)	
High school/tertiary school	66 (39%)	292 (30%)	46 (33%)	
Bachelor’s degree	59 (35%)	346 (36%)	31 (22%)	
Master’s degree and above	5 (2.9%)	50 (5.2%)	3 (2.2%)	
Antenatal anemia	55 (32%)	239 (25%)	26 (19%)	0.025
Advanced age at conception	15 (8.8%)	184 (19%)	27 (20%)	0.004
Chronic hypertension	1 (0.6%)	5 (0.5%)	7 (5.1%)	<0.001
Mode of conception				0.082
Natural	96 (56%)	479 (50%)	70 (51%)	
In vitro fertilization	75 (44%)	478 (50%)	65 (47%)	
Intra Uterine Insemination	0 (0%)	4 (0.4%)	3 (2.2%)	
Cervical Insufficiency	13 (7.6%)	47 (4.9%)	8 (5.8%)	0.30
Gestational age in weeks Median (IQR)	36.71 (35.14, 37.29)	36.71 (35.29, 37.29)	36.57 (35.18, 37.29)	0.40
Sex of the newborns				0.80
Both male	65 (38%)	333 (35%)	50 (36%)	
Both female	53 (31%)	322 (34%)	41 (30%)	
Male and Female	53 (31%)	306 (32%)	47 (34%)	
Number of previous births				0.076
Zero	121 (71%)	632 (66%)	89 (64%)	
One	45 (26%)	270 (28%)	34 (25%)	
Two or more	5 (2.9%)	59 (6.0%)	15 (11%)	
Gestational diabetes mellitus	31 (18%)	241 (25%)	50 (36%)	0.001
Pre-eclampsia	9 (5.3%)	109 (11%)	17 (12%)	0.047
No history of previous preterm birth	171 (100%)	953 (99%)	137 (99%)	0.80

1 Data presented as n (%), unless otherwise stated, IQR- interquartile range

2 Comparison between the three groups was done using Pearson's Chi-squared test; Fisher's exact test; Kruskal-Wallis rank sum test.

**Table-II:** Distribution of outcomes based on maternal pre-pregnancy BMI categories and findings of univariate regression.

Outcomes		N (%)	OR^1^	95% CI^1^	p-value
Low birth weight	Underweight	138 (40.4%)	1.93	1.30, 2.92	0.001
	Overweight or Obese	87 (31.5%)	0.79	0.54, 1.14	0.20
	Normal	658 (34.2%)	Reference		
Intrauterine growth restriction	Underweight	12 (3.5%)	1.53	0.76, 2.87	0.20
	Overweight or Obese	7 (2.5%)	1.09	0.44, 2.31	0.84
	Normal	45 (2.3%)	Reference		
Premature birth	Underweight	95 (27.8%)	1.10	0.79, 1.52	0.58
	Overweight or Obese	78 (28.3%)	1.14	0.80, 1.64	0.48
	Normal	512 (26.6%)	Reference		
Neonatal mortality	Underweight	0 (0%)	--	--	--
	Overweight or Obese	0 (0%)	--	--	--
	Normal	5(0.3%)	Reference		
Low APGAR score at 5 mins	Underweight	0 (0%)	--	--	--
	Overweight or Obese	1 (0.4%)	7.01	0.28, 178	0.17
	Normal	1(0.05%)	Reference		
Admission to NICU	Underweight	104 (30.4%)	1.06	0.76, 1.48	0.73
	Overweight or Obese	89 (32.2%)	1.24	0.86, 1.81	0.26
	Normal	571(29.7%)	Reference		

1 OR = Odds Ratio, CI = Confidence Interval, Odds Ratio is estimated in comparison to Normal pre-pregnancy BMI (Reference), Number of neonates in underweight, normal BMI and overweight/obese categories were 342, 1922 and 276 respectively.

**Table-III:** Findings of multivariable regression for the association between maternal pre-pregnancy BMI and neonatal outcomes in twin pregnancies.

Outcomes		OR^1^	95% CI^1^	p-value
Low birth weight	Underweight	1.88	1.26, 2.89	0.003
	Overweight or Obese	0.80	0.54, 1.18	0.25
	Normal	Reference		
Intrauterine growth restriction	Underweight	1.36	0.66, 2.61	0.38
	Overweight or Obese	1.08	0.43, 2.36	0.86
	Normal	Reference		
Premature birth	Underweight	1.08	0.77, 1.53	0.66
	Overweight or Obese	1.16	0.80, 1.69	0.44
	Normal	Reference		
Neonatal mortality	Underweight	--	--	--
	Overweight or Obese	--	--	--
	Normal	Reference		
Low APGAR score at 5 mins	Underweight	--	--	--
	Overweight or Obese	8.36	0.28, 259	0.17
	Normal	Reference		
Admission to NICU	Underweight	1.03	0.73, 1.46	0.86
	Overweight or Obese	1.17	0.80, 1.73	0.42
	Normal	Reference		

1 OR = Odds Ratio, CI = Confidence Interval, Odds Ratio is estimated in comparison to Normal pre-pregnancy BMI (Reference), Adjusted for maternal education, antenatal anemia, advanced maternal age, chronic hypertension, gestational diabetes mellitus status, and pre-eclampsia.

## DISCUSSION

Our findings show that PPBMI plays a significant role in influencing LBW in twins, particularly in the context of underweight mothers. In contrast, no statistically significant associations were observed for the other neonatal outcomes. Maternal underweight status was identified as a significant risk factor for delivering twins with low birth weight. This finding is consistent with existing literature, suggesting maternal undernutrition may compromise fetal growth and lead to LBW.^23^,^24^ A recent study by Qu P et al analyzed 3,431 twin pregnancies and showed that underweight mothers had twins with lower birth weights, while obese mothers had twins with higher birth weights and increased risk of preterm birth.^25^

Our study had a small sample of obese mothers, which may have limited our ability to detect significant associations between maternal obesity and neonatal outcomes. The physiological demands of twin pregnancies, which already place a significant strain on maternal resources, may exacerbate the effects of insufficient pre-pregnancy weight, leading to low birth weight of offspring. Our results further highlight the importance of adequate maternal nutrition and BMI management before conception. Except for the incidence of LBW, PPBMI in our study did not impact other neonatal outcomes, including IUGR, prematurity, admission to the NICU, and low APGAR scores. Our findings confirm other studies that also showed that maternal BMI had no detectable effect on neonatal outcomes in twin pregnancies.^26^,^27^

The absence of significant associations with IUGR and prematurity is particularly notable, as these outcomes are often closely related to LBW. One possible explanation could be that other factors, such as pregnancy weight gain, overall maternal health, or pregnancy complications, may be more significant in determining these outcomes in twin pregnancies, necessitating further higher-quality research. Similarly, the lack of association with NICU admission and low APGAR scores may reflect the complexity of these outcomes, which are influenced by many factors beyond maternal BMI, including the quality of neonatal care and intrapartum events.

Our study is one of the few to examine the link between maternal pre-pregnancy BMI (PPBMI) and neonatal outcomes in twin pregnancies, revealing that maternal underweight status significantly raises the risk of low birth weight (LBW). This insight provides a clearer understanding of how maternal nutrition, specifically pre-pregnancy BMI, can impact neonatal outcomes in twin pregnancies, which are already at higher risk for complications compared to singleton pregnancies. The findings support the need for targeted nutritional interventions before and during pregnancy to enhance neonatal outcomes in twin deliveries. Given the observed association, healthcare providers should closely monitor and manage the nutritional status of women planning to conceive, particularly those at risk for twin pregnancies. Nutritional counseling and interventions to achieve optimal PPBMI may help reduce the risk of LBW and improve outcomes in this population.

### Strengths:

The strengths of this study are a fairly large sample size, reliable and objective measurement of exposure (maternal BMI) and outcomes, and the use of multivariable regression analysis to control confounders.

### Limitations:

Firstly, we included all participants for whom data was available without calculating a specific sample size. While we think the current sample size of 1270 participants is acceptable, the number of obese women was limited, which required us to combine them with the overweight group. Therefore, an individual association of overweight and obese PPBMI in the categories with neonatal outcomes in twin pregnancy could not be ascertained. Secondly, while we adjusted for some of the potential confounders, it is possible that some variables were not accounted for in the multivariable model. Larger studies with more diverse populations should validate our results and explore pathways linking maternal underweight PPBMI to adverse neonatal outcomes in twin pregnancies.

## CONCLUSION

In twin pregnancies, maternal underweight status pre-pregnancy increases the risk of LBW in neonates. At the same time, pre-pregnancy BMI did not impact other neonatal outcomes, such as IUGR, APGAR scores, or the rates of NICU admission. Our findings highlight the importance of pre-pregnancy nutritional status in optimizing neonatal outcomes in twin deliveries

### Implications for future research:

Further research is needed to explore the mechanisms underlying this observed association between PPBMI and LBW, particularly how other maternal factors, such as gestational weight gain and overall health, may influence this association. Future studies with larger, more diverse populations could help validate our findings. Additionally, follow-up studies on the long-term developmental outcomes, such as growth, metabolic health, and developmental milestones, in twins born to mothers across various BMI categories could offer a more comprehensive understanding of the impact of maternal BMI on the lifelong health of these children.

### Authors’ contributions:

**LZ:** Was involved in concept, the study design and manuscript writing.

**LH, JL, TY, and QL:** Were involved in data collection, analysis, interpretation and did critical review.

**LL:** Was involved in the revision of manuscript and validation.

**LZ, QL, LL:** Helped in obtaining the funding, critical review.

All authors have read, approved the final manuscript and are responsible for the integrity of the study.
